# First Draft Genome for a *Burkholderia mallei* Isolate Originating from a Glanderous Mule from Brazil

**DOI:** 10.1128/genomeA.00579-17

**Published:** 2017-07-13

**Authors:** G. Girault, C. Woudstra, B. Martin, F. Vorimore, V. Lucia de Assis Santana, P. Fach, N. Madani, K. Laroucau

**Affiliations:** a University Paris-Est, Anses, Animal Health Laboratory, Bacterial Zoonoses Unit, Maisons-Alfort, France; b University Paris-Est, Anses, Food Research Laboratory, IdentyPath platform, Maisons-Alfort, France; c Ministry of Agriculture–MAPA, Recife, Pernambuco, Brazil

## Abstract

*Burkholderia mallei* is the etiological agent of glanders. Here, we present the draft genome sequence of *Burkholderia mallei* strain 16-2438_BM#8 that was isolated from a mule found dead in Pernambuco, northeast Brazil. It is the first available genomic sequence from a strain isolated on the American continent.

## GENOME ANNOUNCEMENT

Glanders is an infectious disease of solipeds and humans caused by the Gram-negative bacterium *Burkholderia mallei*. The implementation of glanders control programs in many countries at the beginning of the 20th century led to the eradication of the disease in the United States, Canada, and Europe (1). Glanders is included in the list of the notifiable diseases to report to the World Organization for Animal Health (2). In recent years, several outbreaks of glanders occurred in equine populations in Asia, the Middle East (Afghanistan, Iraq, Iran, Kuwait, Pakistan, Syria, and UAE), Africa, and South America (Brazil). Due to the recent increase of cases in multiple countries, glanders has regained the status of a reemerging disease (1).

Prior to the disease’s reappearance in the northeast of Brazil in 1999 (3), with several cases reported to date (4), the disease was last reported in the state of Rio de Janeiro in 1968.

A *B. mallei* strain (16-2438_BM#8) was isolated in 2016 from a lung tissue collected from a mule found dead in Paudalho, Pernambuco, Brazil. Isolation and identification were realized following the OIE manual (2).

Genomic DNA was extracted from a 48 h culture, incubated at 37°C on nutrient agar containing 4% glycerol, using the DNeasy blood and tissue kit (Qiagen, Hilden, Germany) according to the manufacturer’s instructions for Gram-negative bacteria, with an additional RNase A (Roche) treatment. Libraries were prepared using the Nextera XT kit (Illumina). Whole-genome sequencing was performed using an Illumina MiSeq (Illumina) according to the manufacturer’s instructions. The MiSeq run was carried out on the DNA preparation, with paired-end reads of 150 bp using MiSeq v2 reagents and with a sequencing depth of 170×. The raw reads were trimmed using Trimmomatic-0.36 (phred33, minimum length 50 bp) and assembled *de novo* using SPAdes 3.7.1 (with iterating kmer values of 55, 77, 87, 99, and 121), producing 296 contigs larger than 1,000 pb. MeDuSa was used to generate scaffolds from the contigs and to perform the mapping against *B. mallei* ATCC 23344 as a reference genome (5). A total of 56 scaffolds larger than 1,000 bp were obtained. The total size of the assembly is 5,684,043 bp. The *N*_50_ values of the assembly are 29,740 bp and 170,379 bp for the contigs and scaffolds, respectively. As expected for the genus, the G+C content was high, averaging 68.68%. A total of 4,886 coding sequences have been predicted using QUAST (Quality Assessment Tool for Genome Assemblies).

A detailed comparative genomic analysis of this strain with other *B. mallei* strains from different geographical and host origins will improve the knowledge of this reemerging disease.

### Accession number(s).

This whole-genome sequencing project (PRJEB20536) has been deposited in the European Nucleotide Archive (ENA) (http://www.ebi.ac.uk/ena/data/view/PRJEB20536) under the accession numbers FXLG01000001 to FXLG01000072. The version described in this paper is the first version.
